# Supplementary material for: Non-Destructive Detection of Strawberry Quality Using Multi-Features of Hyperspectral Imaging and Multivariate Methods
Source: Sensors (Basel). 2020 May 29;20(11):3074. doi: 10.3390/s20113074 (PMC7308843; doi:10.3390/s20113074)
Supplement: Supplementary file 1 [file sensors-20-03074-s001.pdf]

Non-Destructive Detection of Strawberry Quality Using Multi-Features of Hyperspectral Imaging and Multivariate Methods

Shizhuang Weng *,†, Shuan Yu †, Binqing Guo, Peipei Tang and Dong Liang

National Engineering Research Center for Agro-Ecological Big Data Analysis & Application, Anhui University, 111 Jiulong Road, Hefei 230601, China; yushuan_1994@163.com(S.Y.); guobingqing65@gmail.com(B.G.); gdsama1101@163.com(P.T); comm_2006@foxmail.com(D.L)

* Correspondence: weng_1989@126.com; Tel.: +86-13695601875

† These authors contributed equally to this work.

Received: 11 May 2020; Accepted: 26 May 2020; Published: 29 May 2020

Supplementary Material

The Vis-NIR hyperspectral imaging system consists of a high spectrograph, a CCD camera, two tungsten halogen lamps, a moving platform, a black box and a computer with the image acquisition software.

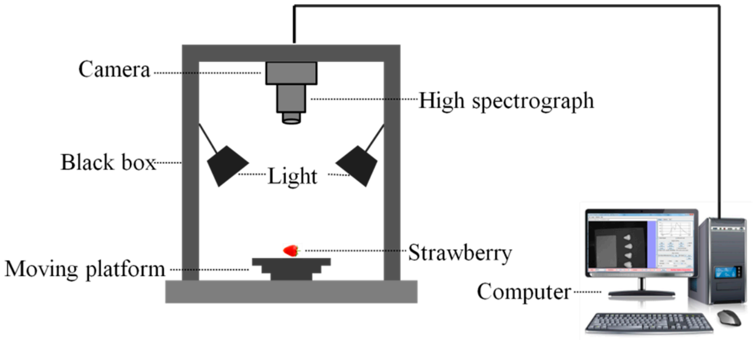

Figure S1. Vis-NIR hyperspectral imaging system.

The calibration curve based on Lambert-Beer law are shown in Figure S2. The VC concentration are calculated by the curve, where y is the absorbance value of VC at 250 nm, and x is the VC concentration.

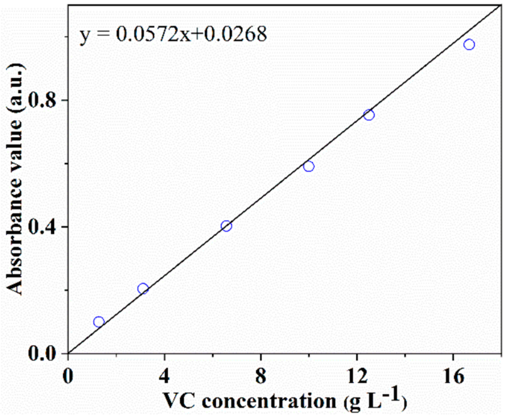

Figure S2. The standard curve for calculating VC concentration.

The spectra preprocessed by WT are shown in Figure S3. From figure, the spectra are smoother than raw spectra and the difference of preprocessed spectra is more obvious.

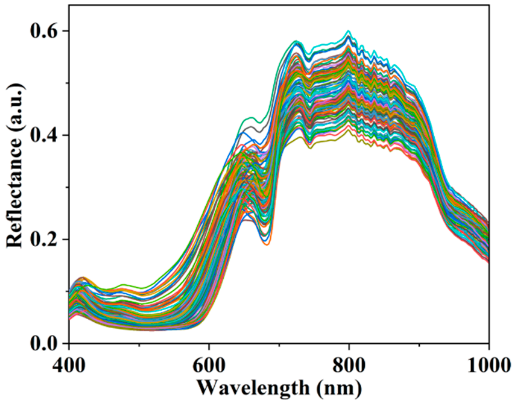

Figure S3. Reflectance spectra of strawberries pretreated by WT.

The normalized color and texture features of first band for strawberries with different pH and VC contents are showed in Figure S4. With variation of pH and VC content, the change trend of values of color features is partially identical, and the data of color features exists redundant. The changes of texture features are diversified, and the information is richer.

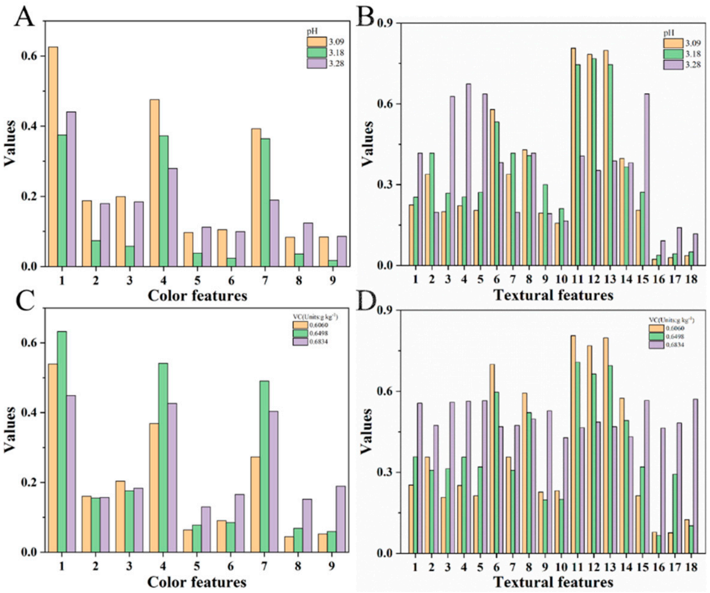

Figure S4. Color and textural features of strawberries with different pH and VC contents. Color (A) and textural (B) features with different pH, color (C) and textural (D) features with different VC contents.

The relationship between the RMSECV and number of variables selected by CARS are show in Figure S5. From figure, the RMSECV curves of Figure S5 A-E both tend to decline in fluctuations. The number of selected variables with the lowest RMSECV for spectroscopy of SSC, pH, VC, color of SSC, and texture of VC are 59, 45, 27, 3 and 23.

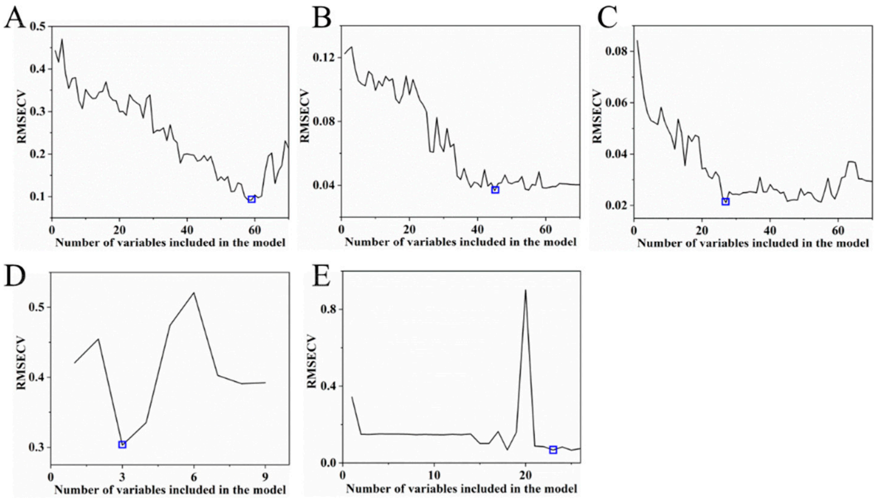

Figure 5. Relationship between the RMSECV and the number of selected variables. Selected spectral variables for SSC (**A**), pH (**B**) and VC (**C**), selected color variables for SSC (**D**) and selected texture variables for VC (**E**).

The concrete wavelengths selected using CARS are shown in Table S1, and the wavelength order is arranged in descending order of weight. The important wavelengths for SSC and pH are distributed in the range of 400–1000 nm, and the selected wavelengths for VC are concentrated in the range except 560–655 nm. The images corresponding to the first two wavelengths of each quality parameter are used to select texture features, and the selected wavelengths were 676 and 910 nm for SSC, 444 and 842 nm for pH, and 837 and 891 nm for VC.

Table S1. Concrete wavelengths by CARS for SSC, pH and VC.

Parameters	Wavelengths (nm)
SSC	676;910;668;785;605;704;489;965;459;553;518;957;650;868;944;671;491;744;980;821;643; 580;806;891;543;873;972;496;999;484;951;645;603;628;949;732;655;925;635;933;630;837; 585;839;548;430; 721;608;452;464;583;610;757;528;508;600;978;513;523
pH	444;842;957;824;811;883;491;496;972;625;401;585;648;565;543;701;980;959;605;673;765; 928;658;986;489;685;806;923;683;650;449;403;742;726;855;978;580;676;545;479;698;603; 873;420;484;
VC	837;891;796;658;936;808;724;432;666;959;471;951;983;801;558;754;442;479;521;526;870; 980;734; 912;706;811;783

Based on spectral features, the results using important variables selected by CARS are shown in Table S2. The prediction performance of pH and VC are higher than the model using full-range spectroscopy, and the accuracy of SSC model is lower. Compared to models of variables of multiple features, the prediction results of SSC and VC are both poorer.

Table S2. Prediction results of SSC, pH and VC of strawberries using important variables of spectral features.

Parameters	Methods	Variables	Setting of parameter	R_c^2	RMSEC	R_p^2	RMSEP
SSC	LWR	46	a = 26, n_neigh = 43, distance = 2, weighting = 2	0.9592	0.0881	0.9342	0.1143
pH	LWR	45	a = 23, n_neigh = 35, distance = 2, weighting = 2	0.9934	0.0447	0.8858	0.0108
VC	PLSR	39	nLVs = 8	0.9486	0.0162	0.8899	0.0265

Parameter settings for multivariate analysis methods for predicting SSC, pH and VC of strawberries were shown in Table S3-6.

Table S3. Parameter setting of multivariate analysis methods using reflectance spectroscopy.

Parameters	Methods	Pretreatment	Setting of Parameters
SSC	PLSR	Raw	nLVs = 12
		WT	nLVs = 12
		MSC	nLVs = 12
	SVR	Raw	linear kernel function, t = 0.01, c = 2048, g = 0.0078
		WT	linear kernel function, t = 0.01, c = 1024, g = 0.031
		MSC	linear kernel function, t = 0.01, c = 2048, g = 0.0078
	LWR	Raw	a = 71, n_neigh = 87, distance = 2, weighting = 2
		WT	a = 50, n_neigh = 90, distance = 2, weighting = 2
		MSC	a = 30, n_neigh = 68, distance = 2, weighting = 2
pH	PLSR	Raw	nLVs = 9
		WT	nLVs = 10
		MSC	nLVs = 12
	SVR	Raw	linear kernel function, t = 0.01, c = 2048, g = 0.0039
		WT	linear kernel function, t = 0.01, c = 2048, g = 0.0039
		MSC	linear kernel function, t = 0.01, c = 2048, g = 0.0156
	LWR	Raw	a = 24, n_neigh = 44, distance = 2, weighting = 2
		WT	a = 47, n_neigh = 64, distance = 2, weighting = 2
		MSC	a = 15, n_neigh = 47, distance = 2, weighting = 2
VC	PLSR	Raw	nLVs = 11
		WT	nLVs = 11
		MSC	nLVs = 12
	SVR	Raw	linear kernel function, t = 0.01, c = 1024, g = 0.0078
		WT	linear kernel function, t = 0.01, c = 1024, g = 0.0078
		MSC	linear kernel function, t = 0.01, c = 2048, g = 0.0156
	LWR	Raw	a = 40, n_neigh = 81, distance = 2, weighting = 2
		WT	a = 66, n_neigh = 83, distance = 2, weighting = 2
		MSC	a = 28, n_neigh = 75, distance = 2, weighting = 2

PLSR: nLVs — number of latent variables.

SVR: t — loss function;

c — loss function parameters;

g — gamma function in the kernel function.

LWR: a — dimensions to consider;

n_neigh — nearest neighbours to consider;

distance — 1=Euclidean, 2=Mahalanobis;

weighting — 1=Uniform, 2=Cubic.

Table S4. Parameter setting of multivariate analysis methods using spectroscopy, color and texture.

Parameters	Methods	Features	Setting of parameters
SSC	PLSR	Spectroscopy+color	nLVs = 12
		Spectroscopy+texture	nLVs = 11
		Spectroscopy+color+texture	nLVs = 12
	SVR	Spectroscopy+color	linear kernel function, t = 0.01, c = 4096, g = 0.00195
		Spectroscopy+texture	linear kernel function, t = 0.01, c = 2048, g = 0.0039
		Spectroscopy+color+texture	linear kernel function, t = 0.01, c = 1024, g = 0.0039
	LWR	Spectroscopy+color	a = 71, n_neigh = 87, distance = 2, weighting = 2
		Spectroscopy+texture	a = 50, n_neigh = 90, distance = 2, weighting = 2
		Spectroscopy+color+texture	a = 41, n_neigh = 82, distance = 2, weighting = 2
pH	PLSR	Spectroscopy+color	nLVs = 11
		Spectroscopy+texture	nLVs = 10
		Spectroscopy+color+texture	nLVs = 10
	SVR	Spectroscopy+color	linear kernel function, t = 0.01, c = 4096, g = 0.00195
		Spectroscopy+texture	linear kernel function, t = 0.01, c = 1024, g = 0.0078
		Spectroscopy+color+texture	linear kernel function, t = 0.01, c = 1024, g = 0.0039
	LWR	Spectroscopy+color	a = 32, n_neigh = 88, distance = 2, weighting = 2
		Spectroscopy+texture	a = 36, n_neigh = 79, distance = 2, weighting = 2
		Spectroscopy+color+texture	a = 37, n_neigh = 81, distance = 2, weighting = 2
VC	PLSR	Spectroscopy+color	nLVs = 9
		Spectroscopy+texture	nLVs = 10
		Spectroscopy+color+texture	nLVs = 10
	SVR	Spectroscopy+color	linear kernel function, t = 0.01, c = 1024, g = 0.0039
		Spectroscopy+texture	linear kernel function, t = 0.01, c = 1024, g = 0.0039
		Spectroscopy+color+texture	linear kernel function, t = 0.01, c = 1024, g = 0.0039
	LWR	Spectroscopy+color	a = 89, n_neigh = 90, distance = 2, weighting = 2
		Spectroscopy+texture	a = 46, n_neigh = 62, distance = 2, weighting = 2
		Spectroscopy+color+texture	a = 41, n_neigh = 84, distance = 2, weighting = 2

Table S5. Parameter setting of CARS and UVE.

Variable Selection	Setting of Parameters
CARS	A = 25, fold = 5, method = 'center', num = 50
UVE	A=25, method='center', N=1000, ratio=0.75

CARS: A — the maximal principle to extract;

fold — the group number for cross validation;

method — pretreatment method;

num — the number of Monte Carlo Sampling runs.

UVE: A — The max principal component for cross-validation;

method — pretreatment method;

N — The number of Monte Carlo Simulation;

ratio — The ratio of calibration samples to the total samples.

Table 6. Parameter setting of multivariate analysis methods based on important variables.

Variable Selection	Parameters	Methods	Setting of Parameters
CARS	SSC	PLSR	nLVs = 9
	pH	LWR	a = 23, n_neigh = 35, distance = 2, weighting = 2
	VC	PLSR	nLVs = 11
UVE	SSC	PLSR	nLVs = 12
	pH	LWR	a = 16, n_neigh = 49, distance = 2, weighting = 2
	VC	PLSR	nLVs = 12
